# Acute febrile neutrophilic dermatosis (Sweet's syndrome) in a child, associated with a rotavirus infection: a case report

**DOI:** 10.1186/1752-1947-4-281

**Published:** 2010-08-20

**Authors:** Alexandros Makis, Stavros Stavrou, Nikolaos Chaliasos, Aikaterini Zioga, Antonios P Vlahos, Georgios Gaitanis, Antigone Siamopoulou, Ioannis D Bassukas

**Affiliations:** 1 Child Health Department, University of Ioannina Medical School, P.O. Box 1187, GR-45110 Ioannina, Greece; 2 Department of Pathology, University Hospital of Ioannina, Niarchou str, GR-45500, Ioannina, Greece; 3 Department of Dermatology and Venereal Diseases, University Hospital of Ioannina, Niarchou str, GR-45500, Ioannina, Greece

## Abstract

**Introduction:**

Sweet's syndrome characterized by fever, blood neutrophilia and inflammatory skin lesions, is rarely diagnosed in children. It presents in three clinical settings: classical Sweet's syndrome, usually after a respiratory tract infection; malignancy-associated, frequently related to acute myelogeneous leukemia; and drug-induced. We present, to the best of our knowledge, the first case of a rotavirus -infection-related Sweet's syndrome.

**Case presentation:**

An 18-month-old boy of Hellenic origin was referred to us with diarrhea, fever, neutrophilia, typical skin lesions, asymmetrical hip arthritis and oropharyngeal involvement. A skin biopsy confirmed the diagnosis. Thorough screening did not reveal any underlying systemic illness, except for the confirmation of an overt rotavirus infection. The syndrome responded promptly upon corticosteroid administration; no recurrence was observed.

**Conclusion:**

Besides describing the connection of Sweet's syndrome to a rotavirus infection, this case report is also a reminder that in a child presenting with a febrile papulo-nodular rash with neutrophilia Sweet's syndrome should be included in the differential.

## Introduction

Sweet's syndrome (acute febrile neutrophilic dermatosis) is characterized by a constellation of clinical symptoms and physical findings, which include fever, blood and tissue neutrophilia, leading to the development of tender, erythematous inflammatory skin lesions (papules, nodules, plaques), histopathologically characterized by the presence of abundant mature neutrophils [1]. It presents in three clinical settings: 'classical' ('para-infectious') Sweet's syndrome, representing a hypersensitivity reaction preceding infection; malignancy-associated ('para-neoplastic') Sweet's syndrome (in children usually associated with acute myelogenous leukemia); and less frequently as an adverse drug reaction, sometimes in connection with certain underlying diseases (drug-induced Sweet's syndrome) [2]. Irrespective of its cause, clinical and laboratory signs in Sweet's syndrome respond promptly to systemic corticosteroids. Spontaneous resolution is possible, although a persisting recurrent course over months is the rule [3].

Sweet's syndrome is rarely diagnosed in children [4]. Here, on the occasion of a child with classical, "parainfectious" Sweet's syndrome we present, to the best of our knowledge for the first time in the accessible literature, the association of this syndrome to a rotavirus infection. The current report appends rotavirus to the list of infectious agents associated with Sweet's syndrome, thus expanding the pertinent diagnostic criteria [5].

## Case presentation

An 18-month-old boy of Hellenic origin was initially admitted to another hospital because of fever up to 39.9°C lasting for five days, a mild cough, one to two vomits and two to three yellowish diarrheas per day. On examination, apart from fever (38.8°C) no other pathological findings were present. Laboratory evaluation revealed leukocytosis (26,500/μl) with 47% neutrophils and an elevated erythrocyte sedimentation rate (ESR) (57 mm/1^st ^hour) and C-reactive protein (CRP) (100 mg/l) (Table 1). Tests for infections were negative except for the detection of a rotavirus antigen (immuno-chromatographic test). The cultures from skin lesions were negative. On day one of hospitalization a papulo-nodular rash with lesions of up to 2 cm in diameter, some of them with central ulceration, appeared on the trunk and extremities. The child remained febrile and in the following days new crops of similar skin lesions erupted, while blood neutrophilia and elevated ESR and CRP persisted. On day six a restriction of the spontaneous movements of the left hip, indicative of underlying arthritis was added to his clinical picture, however, without corresponding pathological findings in X-ray and ultrasound imaging. Because of the clinical suspicion of a staphylococcal infection the child was initially treated with intravenous amoxycilline-clavulanic acid, which was later changed to acyclovir, vancomycin and ceftazidime, considering the possibility of disseminated herpes virus infection with secondary bacterial super-infection of the skin lesions, although the relevant cultures were negative.

**Table 1:** Laboratory findings during the course of the syndrome

Laboratory parameters	5^th ^day of illness (admission to another hospital)	15^th ^day of illness (admission to our hospital)	17^th ^day of illness (initiation of prednisone)	20^th ^day of illness (3^rd ^day on prednisone)	57^th ^day of illness (termination of prednisone)
Leukocytes (/μl)	26,500	25,050	22,770	15,550	13,140
Neutrophils (%)	47	71	74	54	19
Lymphocytes (%)	40	21	18	32	73
Hemoglobin (g/dl)	11.9	9.8	9.2	9.5	14.2
Platelets (/μl)	559,000	986,000	807,000	889,000	491,000
ESR (mm/1^st ^hour)	57	100	102	59	4
CRP (mg/l)	100	30	60	12	3
Blood urea nitrogen (mg/dl)	22	11	13	14	21
Serum creatinine (mg/dl)	0.5	0.4	0.4	0.4	0.5
Serum potassium (meq/l)	4.8	4.3	3.6	3.5	4.5
Serum sodium (meq/l)	137	135	135	135	138
AST (IU/l)	37	46	34	27	42
					
ALT (IU/l)	11	17	21	22	15

**Figure 1:**
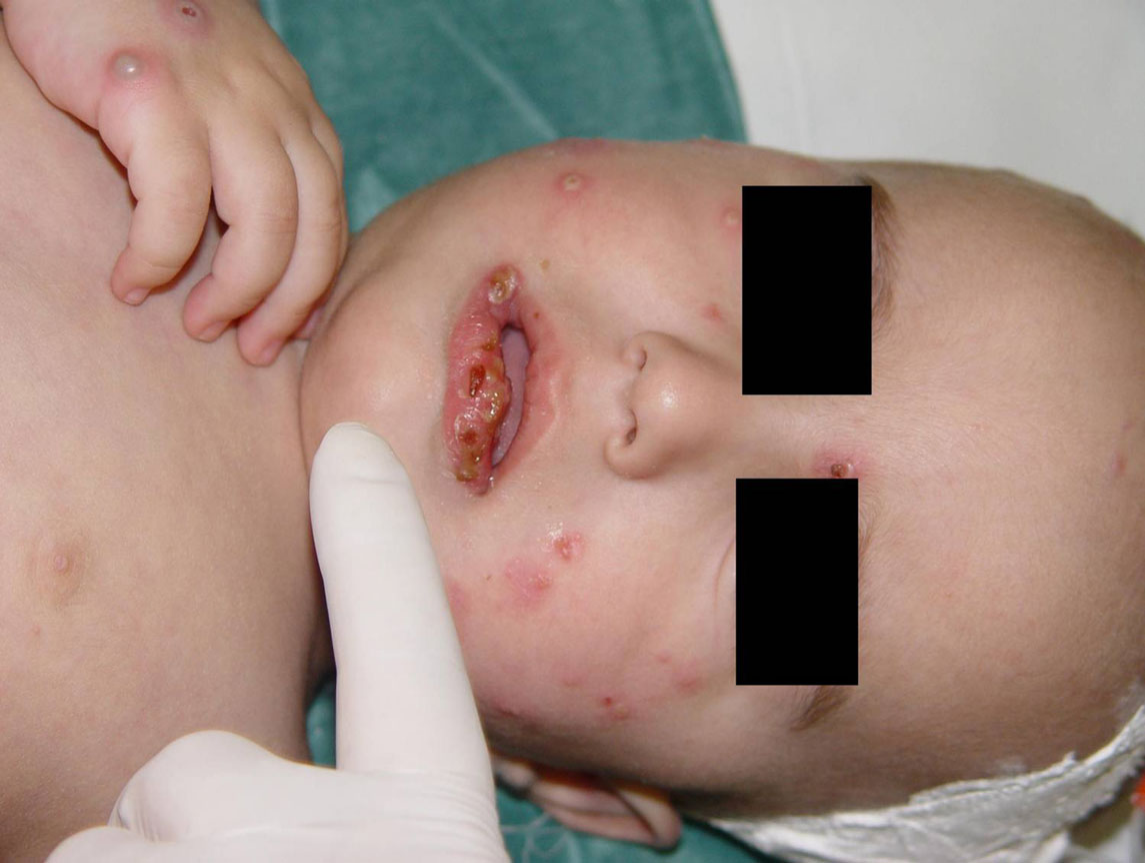
**Papular and nodular-pustular lesions on the face and right hand and partially erosive lips**.

**Figure 2:**
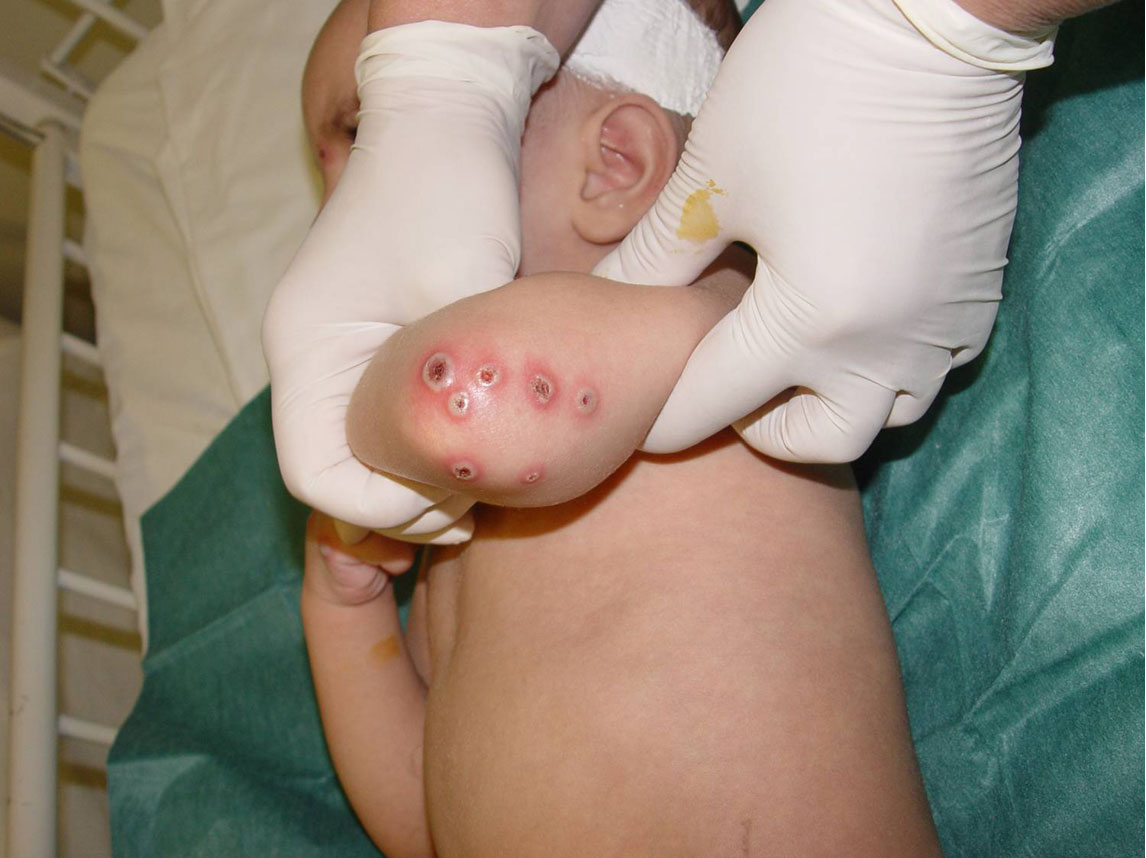
**Centrally ulcerated papular-pustular lesions on the elbow**.

**Figure 3:**
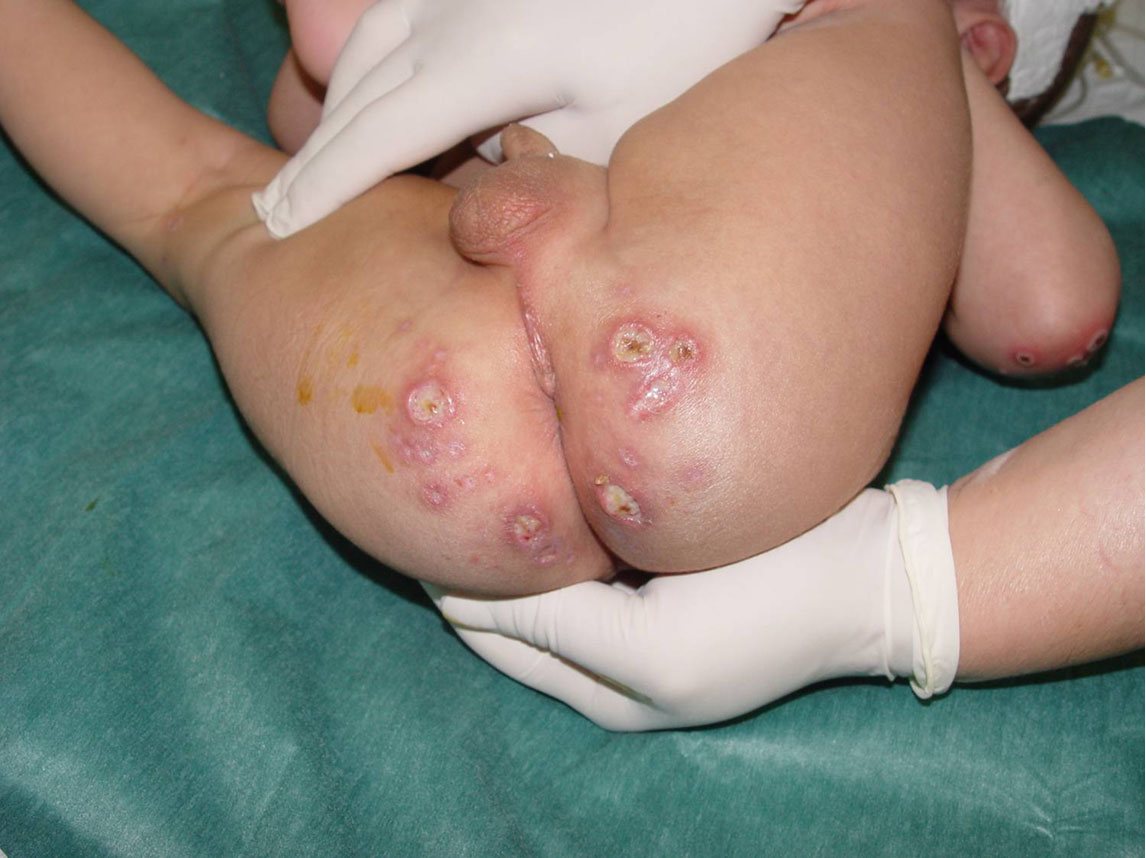
**Almost confluent ulcers on the buttocks**.

On the 11^th ^day of hospitalization, and having shown no substantial improvement, he was transferred to our hospital. On admission he presented with a fever of 39°C, movement restriction of the left hip and multiple, polymorphous skin lesions: deep ulcers, up to about 1 cm in diameter on the buttocks, bullous lesions on the face and extremities, purulent ulcerations on the knees and elbows as well as numerous crusted erosions on the face, extremities, the perianal area and the oropharynx (Figures 1, 2, 3). Blood leukocytosis (25,050/μl) with neutrophilia (71%) and elevated ESR (100 mm/1^st ^hour) and CRP (30 mg/l) were the main laboratory findings (Table 1). Remarkable was the pathergy-like eruption of cutaneous lesions at sites of minimal skin trauma, like intravenous catheter placement. Initial clinical differential diagnoses included Sweet's syndrome as well as a superficial bullous variant of pyoderma gangrenosum, Behcet's disease and bacterial superinfection of varicella lesions. Thus the antibiotic and antiviral treatment was continued and the child was re-evaluated by extensive serological tests for infectious agents and an immunology profile, Tzank smear, microbiological cultures of lesional tissue samples and skin lesion biopsy. On the second day in our department stridor suddenly appeared, an X-ray revealed an airway constriction and intravenous dexamethasone (0.6 mg/kg body weight) was immediately administered. The child responded promptly with improvement of the fever, of the stridor and of his general condition.

**Figure 4:**
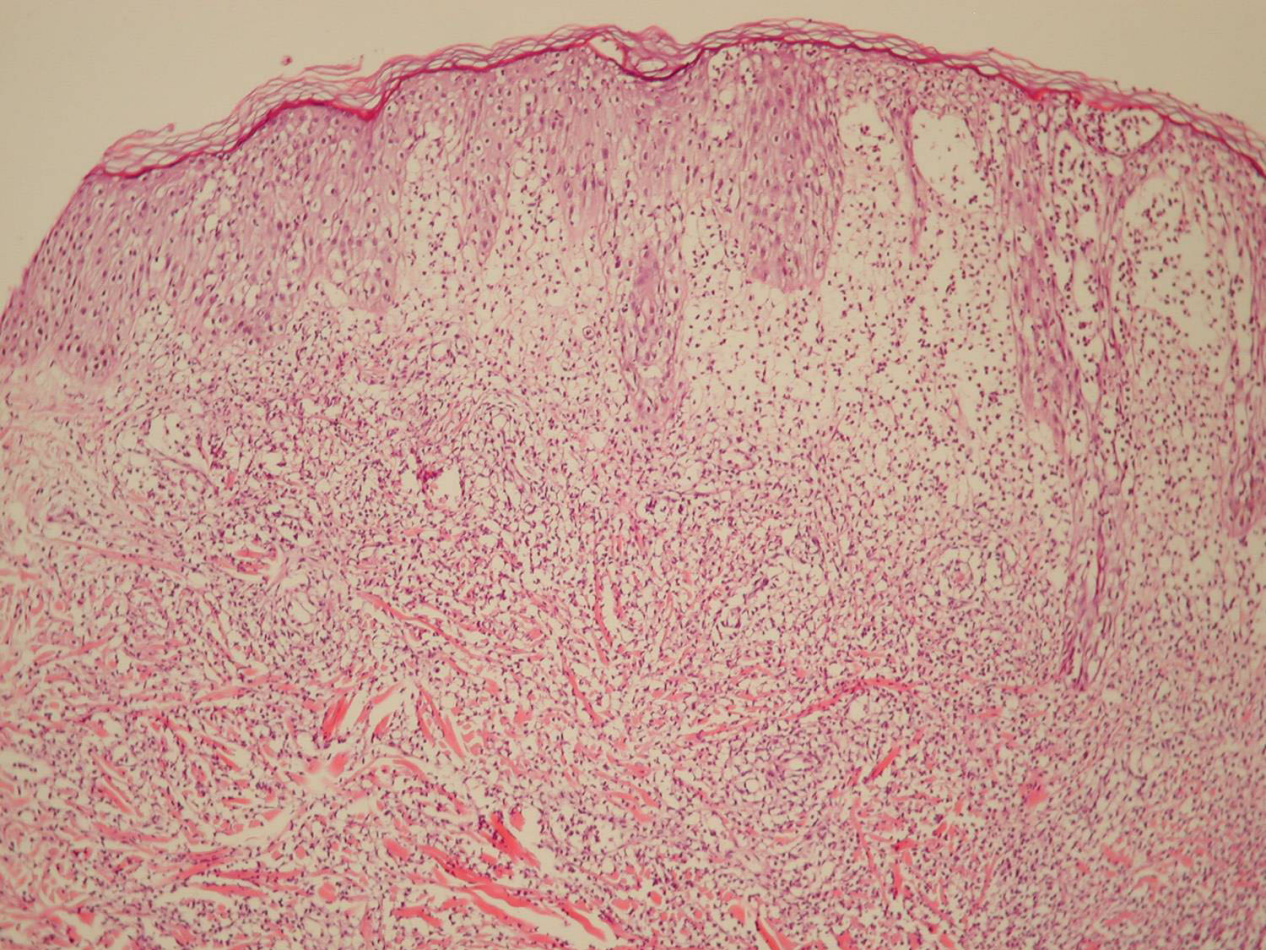
**Histopathology of a skin lesion**. Spongiotic epidermis and exocytosis of neutrophils with focally confluence into microabcesses. Intense edema of the upper dermis and a dense neutrophilic inflammatory infiltrate of the lower dermis. (Hematoxylin-Eosin, × 100).

Skin lesion biopsy revealed a dense inflammatory infiltration of the dermis consisting mainly of mature neutrophils and an intense edema on the upper dermis, findings consistent with the clinical differential diagnosis of Sweet's syndrome (Figure 4). Of the rest of the laboratory evaluations, besides the serological confirmation of a recent rotavirus infection (immuno-chromatographic test), all tests were either negative or within normal ranges. Based on the clinical and laboratory evidence as well as the immediate response to the treatment with corticosteroids the diagnosis of Sweet's syndrome was established (Appendix). The vesicobullous features of the rash, which are not common presentations in Sweet's syndrome, led the differential diagnosis to a superficial bullous variant of pyoderma gangrenosum, probably associated with an emerging hematological malignancy [3]. Therefore, a screening for a concomitant malignancy (bone marrow aspirate and trephine, chest X-ray and an abdominal ultrasound) was performed with no abnormal findings.

Antibiotic and antiviral medications were discontinued and intravenous prednisone (1 mg/kg body weight/24 h) was administered for 10 days with immediate cessation of the fever and gradual improvement of the skin lesions, the hip complaints and the laboratory findings (Table 1). Thereafter, prednisone was continued orally and tapered off slowly over a period of 40 days. After six months of follow-up no recurrence has been observed and the child is thriving.

## Discussion

We report on the case of an atypical vesicobullous variant of Sweet's syndrome in a child with a serologically documented preceding rotavirus infection. Although gastroenteritis often precedes an 'idiopathic' Sweet's syndrome, this is the first, to the best of our knowledge, documented case of an associated rotavirus infection and this syndrome. Given (a) the high prevalence of rotavirus infections, (b) the frequency of gastroenteritis as a concomitant disease of the Sweet's syndrome and (c) the wide suspicion in the literature that this syndrome is still under-diagnosed, this is a remarkable discrepancy, which calls for further exploration in future studies.

Sweet's syndrome was originally described in 1964 by Douglas Sweet as an 'acute febrile neutrophilic dermatosis' [1]. Since then several cases have been reported in the literature, most of them in adults but also in children of all age groups [6]. In children the syndrome is usually of the classic, 'para-infectious' subtype and in most cases follows a respiratory or a gastrointestinal infection. The most frequent nosologic entities that have been associated with this syndrome in children are shown in the Appendix.

Classical or idiopathic ('para-infectious') Sweet's syndrome is characterized by fever (higher than 38°C), blood leukocytosis and neutrophilia, which can precede the cutaneous manifestations for several days, characteristic skin lesions (tender erythematous papules or nodules which can develop into erythematous plaques with a characteristic papillomatous surface). Skin biopsy is the laboratory test that usually confirms the clinical diagnosis of Sweet's syndrome. Edema and a diffuse and perivascularly attenuated inflammatory infiltrate of mature neutrophils with neutrophil fragmentation in the upper dermis, yet without microscopical evidence of vasculitis are the hallmarks of the histopathological picture [7]. The most often affected areas are the upper extremities, the face and the neck. Sometimes the lesions may resemble blisters, while less often, may mimic lesions of pyoderma gangrenosum (Figure 2). Another characteristic clinical feature is the Köbner phenomenon, that is, appearance of 'specific' skin lesions at sites of minor cutaneous trauma (biopsy, venipuncture, and so on.). Skin lesions in Sweet's syndrome usually resolve without scarring.

Apart from the involvement of the integument, as a result of an extensive, multiorganic, sterile neutrophilic inflammatory process other organic systems can be involved as well, as the hip involvement in our patient, probably the result of a sterile arthritis [8]. Also, mucosal involvement is not unusual; it presents as edematous and aphthous lesions of the upper aero-digestive tract in the mouth and pharynx that can lead to airway obstruction, as happened to our patient too [9]. Systemic symptoms that may coexist are headache, myalgias and arthralgias. The syndrome may resolve either automatically or after medication. Recurrence is considered as the most common complication and can take place in various time points. Quite typical is the immediate response to systemic corticosteroids, though after tapering off it recurs in at least one third of the cases. Corticosteroids (prednisone, 1 mg/kg body weight/24 h) are the treatment of choice, usually administered for 10 days and then tapered down slowly to avoid recurrence. However, in children the syndrome is considered more resistant to corticosteroids than in adults and sometimes protracted treatment for up to five months is required in order to avoid recurrences [10]. Topical corticosteroids can be used in patients with localized lesions either as monotherapy or concurrently to systemic prednisone. Potassium iodide and colchicine have also been used with good results as monotherapies or in combination with corticosteroids, while indomethacin, clofazimine, cyclosporine and dapsone are considered as second-line modalities [11]. In malignancy associated cases cure or remission of the underlying disease is followed by the clearance of the symptoms, while in drug induced cases spontaneous improvement is achieved by stopping the suspected medication.

The pathogenesis of Sweet's syndrome is still unknown. Its epidemiological characteristics and the related conditions (infections, malignancies, systemic autoimmune conditions, inflammatory bowel diseases and female predominance) classify this disorder among the diseases that relate to hypersensitivity reactions to bacterial, viral or tumor antigens or to drugs. Circulating autoantibodies, immune complexes and aberrant expression of different cytokines have all been postulated to explain the pathogenesis of this syndrome [12,13]. According to the most recent view, the syndrome reflects a skin-confined or systemic disorder of the homeostasis of the cytokine network leading to abundance of pro-inflammatory cytokines in target tissues, especially of IL-1, G-CSF, GM-CSF and IFN-a. It is worth mentioning that rotavirus infections elicit an increased cytokine production in the intestinal epithelium, especially IL-8 and GM-CSF, which could explain the way by which this infection probably contributed to the induction of the Sweet's syndrome in our patient [14].

## Conclusions

In conclusion, besides reporting the association between a rotavirus infection and Sweet's syndrome in a child, our case report is also a reminder that the differential diagnosis of a febrile child with a papulo-nodular rash and blood neutrophilia should always include Sweet's syndrome. In such cases thorough search for common infectious causes and complete examination for extracutaneous manifestations are mandatory and, after confirming the diagnosis, a step-by-step examination to rule out underlying malignancy is warranted. We think that our report of the correlation of the syndrome with rotavirus infection is important, as isolation of specific causative agents may help in revealing the pathophysiology of this probably underdiagnosed syndrome.

## Appendix

• *Diagnostic criteria for classical Sweet's syndrome (originally proposed by Su and Liu in 1986 and modified by von den Driesch in 1994) *[4,5]. For the establishment of the diagnosis of the syndrome two major and two of the four minor criteria are required. *Major criteria*: 1. Abrupt onset of tender erythematous plaques or nodules; 2. Dense neutrophilic infiltrate of the dermis without leukocytoclastic vasculitis. *Minor criteria*: 1. Fever of over 38°C; 2. Preceding respiratory or gastrointestinal infection or vaccination or association with an hematologic or another malignancy, inflammatory disease or pregnancy; 3. Three of the following four laboratory findings ESR > 20 mm/1^st ^hour, leukocytes > 8.000/μl, neutrophils > 70%, positive CRP; 4. Excellent response to systemic corticosteroids or potassium iodide.

• *Conditions associated with Sweet's syndrome in children*: upper respiratory tract illness, gastrontestinal infection, acute myelogenous leukemia, acute lymphoblastic leukemia, myelodysplastic syndrome, Fanconi's aplastic anaemia, congenital dyserythropoietic anaemia, aseptic meningitis, Behchet's disease, Takayasu arteriitis, systemic lupus erythematosus, ulcerative colitis, T-cell or humoral immunodeficiency, human immunodeficiency virus infection, chronic granulomatous disease, drugs (G-CSF, retinoic acid).

## Competing interests

The authors declare that they have no competing interests.

## Authors' contributions

AM analyzed and interpreted the patient data and wrote the manuscript. SS was a major contributor in writing the manuscript. NC analyzed the patient data regarding the hematological and infection problems. AZ performed the histological examination of the skin. APV contributed in the writing of the manuscript. GG contributed in the differential diagnosis and interpretation of the data. AS supervised the patient's clinical course and contributed to the differential diagnosis and interpretation of the data. IDB supervised the patient's clinical course and was a major contributor in writing the manuscript. All authors read and approved the final manuscript

## Consent

Written informed consent was obtained from the parents of the patient for publication of this case report and accompanying images. A copy of the written consent is available for review by the Editor-in-Chief of this journal.

## References

[B1] Sweet RD An acute febrile neutrophilic dermatosis Br J Dermatol 1964 76 349 356 10.1111/j.1365-2133.1964.tb14541.x 14201182

[B2] Cohen PR Sweet's syndrome--a comprehensive review of an acute febrile neutrophilic dermatosis Orphanet J Rare Dis 2007 2 34 10.1186/1750-1172-2-34 17655751 PMC1963326

[B3] von den Driesch P Sweet's syndrome (acute febrile neutrophilic dermatosis) J Am Acad Dermatol 31 535 556 quiz 557-560 10.1016/S0190-9622(94)70215-2 8089280

[B4] Hospach T von den Driesch P Dannecker GE Acute febrile neutrophilic dermatosis (Sweet's syndrome) in childhood and adolescence: two new patients and review of the literature on associated diseases Eur J Pediatr 2009 168 1 9 10.1007/s00431-008-0812-0 18830624

[B5] Su WP Liu HN Diagnostic criteria for Sweet's syndrome Cutis 1986 37 167 174 3514153

[B6] Halpern J Salim A Pediatric sweet syndrome: case report and literature review Pediatr Dermatol 2009 26 452 457 10.1111/j.1525-1470.2009.00952.x 19689524

[B7] Farhi D Wallach D The neutrophilic dermatoses Dermatol Nurs 2008 20 274 276 279-282 18819221

[B8] Watanabe T Nakashima K Shindo M Yoshida Y Yamamoto O Multiorgan involvement in Sweet's syndrome Clin Exp Dermatol 2009 34 e343 344 10.1111/j.1365-2230.2009.03290.x 19456764

[B9] Bouw J Kater AP van Tongeren J Schultz MJ Upper-airway obstruction instigated by Sweet's syndrome Med Sci Monit 2007 13 CS53 55 17392656

[B10] Cohen PR Neutrophilic dermatoses: a review of current treatment options Am J Clin Dermatol 2009 10 301 312 10.2165/11310730-000000000-00000 19658442

[B11] Yi S Bhate C Schwartz RA Sweet's syndrome: an update and review G Ital Dermatol Venereol 2009 144 603 612 19834438

[B12] Reuss-Borst MA Pawelec G Saal JG Horny HP Muller CA Waller HD Sweet's syndrome associated with myelodysplasia: possible role of cytokines in the pathogenesis of the disease Br J Haematol 1993 84 356 358 10.1111/j.1365-2141.1993.tb03083.x 7691149

[B13] Giasuddin AS El-Orfi AH Ziu MM El-Barnawi NY Sweet's syndrome: is the pathogenesis mediated by helper T cell type 1 cytokines? J Am Acad Dermatol 1998 39 940 943 10.1016/S0190-9622(98)70266-X 9843005

[B14] Ramig RF Pathogenesis of intestinal and systemic rotavirus infection JVirol 2004 78 10213 10220 10.1128/JVI.78.19.10213-10220.2004 PMC516399 15367586

